# Anthra[1,2-*d*][1,2,3]triazine-4,7,12(3*H*)-triones as a New Class of Antistaphylococcal Agents: Synthesis and Biological Evaluation

**DOI:** 10.3390/molecules24244581

**Published:** 2019-12-13

**Authors:** Viktor Zvarych, Maryna Stasevych, Volodymyr Novikov, Eduard Rusanov, Mykhailo Vovk, Piotr Szweda, Katarzyna Grecka, Slawomir Milewski

**Affiliations:** 1 Department of Technology of Biologically Active Substances, Pharmacy and Biotechnology, Lviv Politechnic National University, 13, 79013 Lviv, Ukraine; 2 Department of Mechanism of Organic Reactions, Institute of Organic Chemistry of National Academy of Sciences of Ukraine, 02660 Kyiv, Ukraine; 3 Department of Pharmaceutical Technology and Biochemistry, Gdańsk University of Technology, 80-233 Gdańsk, Poland

**Keywords:** anthra[1,2-*d*][1,2,3]triazine-4,7,12(3*H*)-triones, anti-*Staphylococcusaureus* agents, antibacterial study

## Abstract

The development and spread of resistance of human pathogenic bacteria to the action of commonly used antibacterial drugs is one of the key problems in modern medicine. One of the especially dangerous and easily developing antibiotic resistant bacterial species is *Staphylococcus aureus.* Anthra[1,2-*d*][1,2,3]triazine-4,7,12(3*H*)-triones **22**–**38** have been developed as novel effective antistaphylococcal agents. These compounds have been obtained by sequential conversion of 1-amino-9,10-dioxo-9,10-dihydroanthracene-2-carboxylic acid (**1**) and 1-amino-4-bromo-9,10-dioxo-9,10-dihydroanthracene-2-carboxylic acid (**2**) into the corresponding amides **5**–**21**, followed by subsequent endo-cyclization under the influence of sodium nitrite in acetic acid. Evaluation of the antimicrobial activity of the synthesized compounds against selected species of Gram-positive and Gram-negative bacteria as well as pathogenic yeasts of the *Candida* genus has been carried out by the serial dilution method. It has been established that anthra[1,2-*d*][1,2,3]triazine-4,7,12(3*H*)-triones exhibit selective antibacterial activity against Gram-positive bacteria. Eight, six and seven, out of seventeen compounds tested, effectively inhibited the growth of *S. aureus* ATCC 25923, *S. aureus* ATCC 29213 and *S. epidermidis* ATCC12228, respectively, at a concentration equal to 1 µg/mL or lower. The high antistaphylococcal potential of the most active compounds has been also confirmed against clinical isolates of *S. aureus*, including the MRSA strains. However, bacteria of the *Staphylococcus* genus have demonstrated apparent resistance to the novel compounds when grown as a biofilm. None of the four selected compounds **3234** and **36** at a concentration of 64 µg/mL (128 or 256 × MIC—against planktonic cells) has caused any decrease in the metabolic activity of the staphylococcal cells forming the biofilm. The kinetic time–kill assay revealed some important differences in the activity of these substances. Compound **33** is bacteriostatic, while the other three demonstrate bactericidal activity.

## 1. Introduction

The resistance of human pathogenic bacteria and yeasts to the well-known antibiotics and synthetic chemotherapeutics is becoming a major global health challenge [1,2,3]. Among the array of various antibiotic-resistant pathogenic bacteria, staphylococci belong to the most common and dangerous. They are able to produce a wide array of virulence factors and have evolved mechanisms of resistance to a plethora of antibiotics currently in use for human and animal therapies. As a consequence, bacteria of the *Staphylococcus* genus, particularly *S. aureus*, are responsible for a broad spectrum of difficult to treat diseases including skin and ocular infections, foodborne illness, pneumonia, meningitis, endocarditis, and osteomyelitis [4]. Over the past decades, this bacterium has become the main cause of bloodstream infections (BSI) in the world, with a mortality level of 20–40% [5]. Recent advances in the developments of antistaphylococcal agents are presented in works on search of new non-antibiotic agents: peptidoglycan hydrolases compounds of plant origin (plant extracts, essential oils and their components) [6,7,8,9], vaccines development [8], silver nanoparticles [10], bacteriocins [11], and bacteriophages [12]. Currently, the most reasonable approach for understanding the crucial cellular pathways by which unknown to date (or not used as antimicrobial agents) molecules affect pathogenic microorganisms at a molecular level seems to be transcriptome analysis. Recently, Chauhan and coworkers [13] successfully applied RNA sequencing for the determination of antistaphylococcal modes of action of novel discovered benzimidazole molecules. Comparative analysis of differentially expressed genes between control and treated *S. aureus* cells showed selective hits. Therefore, the search and development for new antibacterial agents effective against these pathogenic bacteria remain an urgent task of the present.

Derivatives of the 9,10-anthraquinone of both natural and synthetic origin are well known for their wide range of biological effects. Of particular interest, compounds with an antibacterial effect are known among them [14,15,16,17]. The 1,2,3-triazinone ring is an important biophore fragment for the creation of new promising antimicrobial agents based on the 9,10-anthraquinone platform. This pharmacophore is a structural component of many natural and synthetic biologically active compounds with various therapeutic effects [18,19]. The compounds with a sedative, diuretic, anesthetic, antiarthritic, antitumor, antibacterial, antifungal, antituberculosis, antiviral, antiinflammatory, antihistamine activity were found among 1,2,3-triazine derivatives connected with various carbo- and heterocyclic fragments [20,21,22,23].

It is known that diazotization of the corresponding *ortho*-amino amides with NaNO_2_ or *t*-BuONO under slightly acidic conditions is one of the most common methods for the formation of the 1,2,3-triazin-4-one ring [15]. Despite the widespread use of this method, condensed carbocyclic 1,2,3-triazin-4-one derivatives are known only for the benzene and naphthalene systems [24]. In the context of the above, herein we report a method for the synthesis of new triazine-containing anthraquinone structures and the study their antibacterial effect for identifying potential antimicrobial agents.

## 2. Results and Discussion

### 2.1. Chemistry

**Scheme 1:**
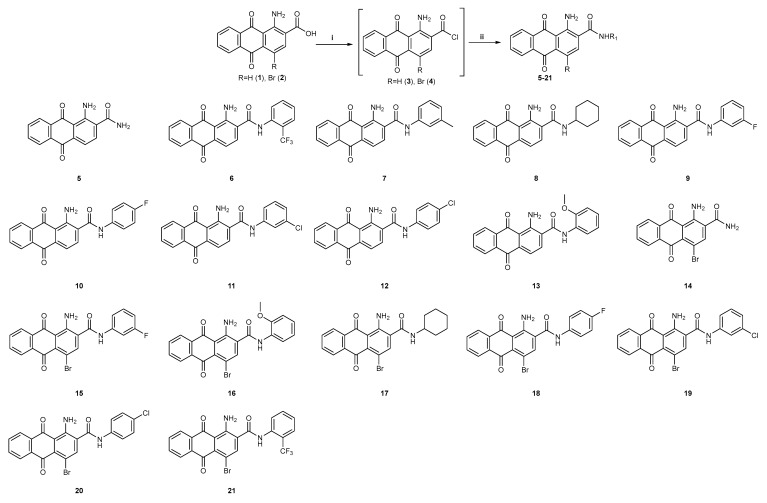
Synthesis of amides **5**–**21**. Reagents and conditions: (i) thionyl chloride (3 eguiv), pyridine (0.15 equiv), dry benzene, 80 °C, 5 h, (ii) for compounds **5** and **14**—25% NH_4_OH (50 mL), −15 °C, 1 h → 80 °C, 3 h → r.t., 10% HCl; for compounds **6**–**13**, **15**–**21**—R_1_NH_2_ (1.1 equiv), dry benzene, TEA (1.1 equiv), ∆, 12 h.

Continuing our research on the directed functionalization of the 9,10-anthraquinone system and the search for new biologically active substances [25,26,27,28,29,30], in the work presented herein we report an effective synthetic approach to a number of new condensed anthraquinone derivatives, namely anthra [1,2-*d*][1,2,3]triazine-4,7,12(3*H*)-triones **22**–**38** (Scheme 1).

Commercially available 1-amino-9,10-dioxo-9,10-dihydroanthracene-2-carboxylic acid (**1**) and 1-amino-4-bromo-9,10-dioxo-9,10-dihydroanthracene-2-carboxylic acid (**2**) were converted into the corresponding acid chlorides **3** and **4** by treatment with thionyl chloride in the presence of a catalytic amount of pyridine. The amides **5**–**21** were prepared from acid chlorides **3** and **4** and ammonia, cyclohexylamine and a variety of anilines (Scheme 1).

**Scheme 2:**
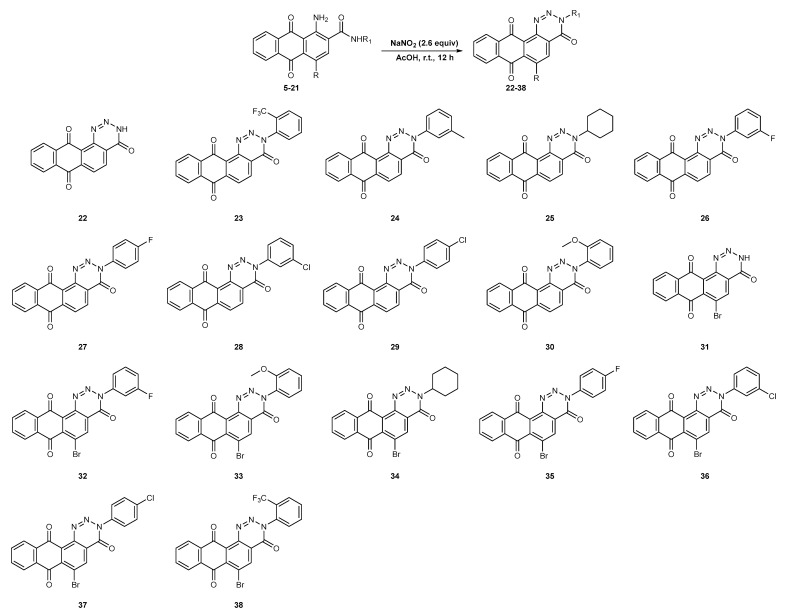
Synthesis of anthra[1,2-*d*][1,2,3]triazine-4,7,12(3*H*)-triones **22**–**38**.

The target compounds **22**–**38** were synthesized via an intramolecular cyclization reaction of amides **5**–**21** with nitrous acid generated in situ in acetic acid at room temperature in 63–90% yields (Scheme 2). It should be noted that the use of *t*-BuONO as a diazotizing agent in the reactions at 20–65 °C proved to be ineffective and gave a low conversion of amides **5**–**21**. Moreover, the presence of a bromine atom at the position 4 of the anthracenedione ring did not provide for a significant impact on the cyclization process.

The formation of amides **6**,**7**,**9**,**10**,**15**,**16**,**18** was confirmed by the presence of a broad singlet of proton of the NH group of the amide fragment in the range of 10.03–10.72 ppm in the ^1^H-NMR spectra. Also, the signal of protons of the NH_2_ group falls into the region of aromatic anthracenedione and benzene protons. The amide protons in *N*-cyclohexyl-substituted amides **8** and **17** resonated in the aromatic proton region of the quinoid ring.

The carbon atom signal of the carbonyl group of amide residue of **5**–**21** was observed within the range of 165.25–169.76 ppm in the ^13^C-NMR spectra. In the case of fluorine-containing amides **9**,**10**,**15**,**18,** the characteristic doublet signals attributable to the C-F bond in the *meta* or *para* position of the benzene residue were presented in the range from 158.19 to 163.45 ppm with a coupling constant of *J* = 241.1 Hz.

**Figure 1:**
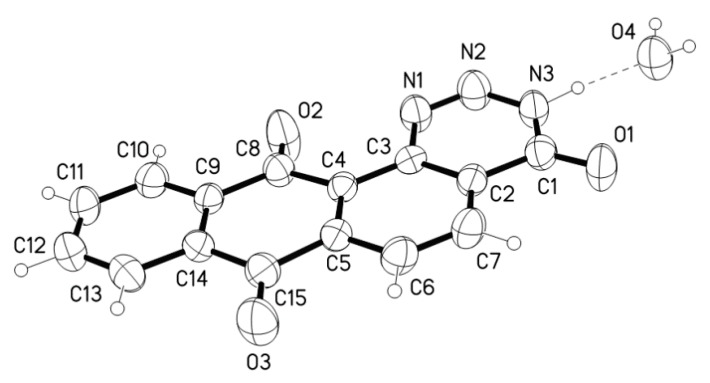
Molecular structure of **22**. Ellipsoids are drawn at 50% probability level (CCDC 1966785).

The characteristic broad singlet signal of NH proton at 15.37 and 15.45 ppm was presented in the ^1^H-NMR spectra of anthratriazinones **22** and **31**. In the ^13^C-NMR spectra of compounds **22**,**31**, the signal of the carbon atom of the carbonyl group in the triazine ring was shifted by 16 ppm compared to the C=O group in the corresponding amides **5** and **14**. Additionally, the structure of anthra[1,2-*d*][1,2,3]triazine-4,7,12(3*H*)-trione **22** was confirmed by X-ray diffraction analysis (Figure 1).

**Figure 2:**
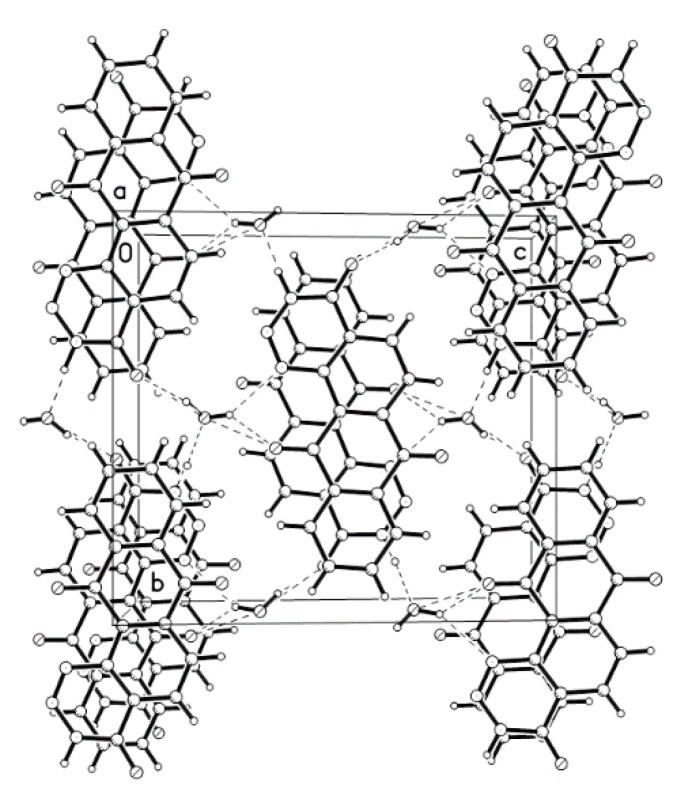
Molecular packing of **22** (viewed along the *a* crystallographic axis).

In structure of compound **22** (Figure 2) the central tetracyclic system is strongly conjugated and planar (Rms deviation of fitted atoms 0.0399), while the atoms O_2_ and O_3_ deviate out of this plane for 0.200(3) and 0.154(3) Å respectively. In crystal molecules packed in columns directed along *a* crystallographic axis.

The shortest intermolecular distances between neighboring molecules in columns are 3.342 Å and 3.424 Å for C···N and C···C contacts correspondingly, thus π-π stacking in columns is observed. Solvate water molecules occupy volume between the columns and bonding together neighboring columns by the system of hydrogen bonds. The parameters of H-bonds in a crystal are shown in Table 1. The combination of π-π stacking in columns along axes *a* and 2D hydrogen bonds across *b* and *c* direction giving 3D net and densely packed crystal with density 1.59 g/cm^3^.

### 2.2. Biological Studies

#### 2.2.1. Determination of MIC Values of Synthesized Agents against Reference Strains of Bacteria and Yeasts and Clinical Isolates of *S. aureus*

The outcomes of our investigation revealed that synthesized anthra[1,2-*d*][1,2,3]triazine-4,7,12(3*H*)-triones exhibited selective antibacterial activity against staphylococci (Table 2). Eight, six and seven out of seventeenths compounds tested inhibited the growth of *S. aureus* ATCC 25923, *S. aureus* ATCC 29,213 and *S. epidermidis* respectively at a concentration equal to 1 µg/mL or lower. This level of activity is comparable to the activity of many antibacterial antibiotics (Table 2) [7,31,32]. The tested antibiotics exhibited activity with the range of MIC from 0.125 to 16.0 µg/mL in the case of staphylococci.

Up to the concentration of 32 µg/mL, no activity was observed against both investigated reference strains of Gram-negative bacteria, namely *E. coli* ATCC 25,922 and *P. aeruginosa* ATCC 27,853 nor the human pathogenic yeasts of the *Candida* genus. Our research revealed also lower activity of investigated antibiotics against Gram-negative bacteria. Six out of seven tested antibiotics did not affect *E. coli* and *P. aeruginosa* up to the highest tested concentration—32.0 µg/mL. The high antistaphylococcal potential of selected agents was additionally confirmed against clinical isolates of *S. aureus*. Interestingly, no difference was observed in the susceptibility of MRSA and MSSA strains (Table 3), which is very important from the clinical point of view. Five of these substances inhibited the growth of all strains tested at the concentration not higher than 1 µg/mL. The four most active compounds, namely **32**–**34**, and **36** were selected for further tests.

Several research groups also synthesized heterocyclic compounds of similar structures. However, according to our best knowledge, none of those compounds exhibited comparable antistaphylococcal activity (MIC values below 1 µg/mL) [33,34,35,36]. Moreover, the results presented by other authors did not indicate similar selectivity of antimicrobial properties (high activity against Gram-positive bacteria and no activity against both Gram-negative bacteria and human pathogenic yeasts) [33,34,35]. In some cases, it is difficult to compare results, because different method of determination of antimicrobial activity (disc diffusion) was used by the authors [33,34].

#### 2.2.2. Bactericidal Effect of Selected Compounds

**Figure 3:**
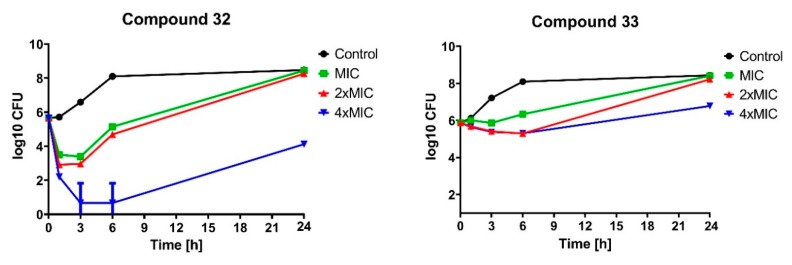

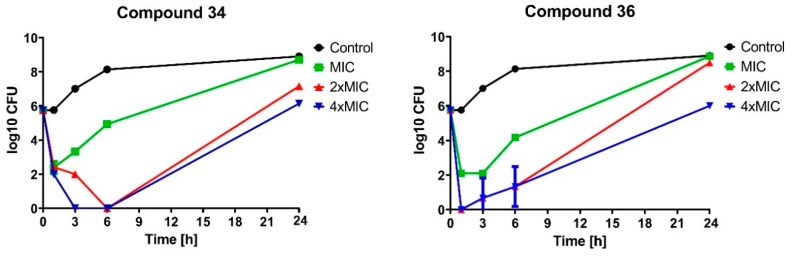
Results of the kinetic time—kill assay for compounds **32**–**34** and **36**.

The structures of four compounds selected for further analysis, namely **32**, **33**, **34** and **36**, are similar. Despite this fact, the kinetic time—kill assay revealed some important differences in the activity of these substances. The respective curves are shown in Figure 3. Compound **33** was apparently bacteriostatic only and did not exhibit any bactericidal effect, while the other three demonstrated a clear such effect, i.e., their action resulted in some reduction of the cell number, even in the case of its concentrations 2 or 4 times higher than MIC. Undoubtedly, compound **34** was the most effective in this respect. A substantial reduction of the log_10_ CFU value (from 6 to 0) of the bacterial cell suspension was observed at 4 × MIC after 1 h-long treatment with this compound and at 2 × MIC after 3 h-long treatment. In the case of compound **36**, such reduction of the cell number was achieved only at 4 × MIC after 1 h-long treatment. The action of compound **32** resulted in a maximal reduction of log_10_ CFU from 6 to 2 after 6 h-long treatment at 4 × MIC. Therefore, the bactericidal potential of compounds tested ranks them in the **34** > **36** > **32** order, whereas compound **33** was not bactericidal at all. For all four compounds, the re-growth of bacterial cells was observed after 24 h-long treatment. This observation indicates that even in the cases when the reduction of log_10_ CFU from 6 to 0 was noted after shorter treatments, some residual live cells must have been preserved.

#### 2.2.3. Activity of the Selected Compounds against Staphylococcal Biofilm

When the *S. aureus* cells were grown in the biofilm mode, the antistaphylococcal potential of compounds **32**–**34** and **36** appeared much lower, if any, than that found against the cells grown in the planktonic mode. None of these compounds caused any decrease in the metabolic activity of the cells forming the biofilm, as measured with the MTT assay, at concentrations as high as 64 µg/mL (128 or 256 × MIC). It is not especially surprising, since many earlier reports indicated that for different antibacterial agents, their effective concentrations inhibiting metabolic activity of staphylococci grown in the biofilm mode can be 10–1000 times higher than those inhibiting the planktonic growth [37]. This is because the embedded bacterial cells are getting an optimal defense mechanism against the adverse effects of antibiotics and the immune system of the host [37]. Thus in our opinion, the evidentially lower activity against biofilm should not be considered as a crucial disadvantage of the synthesized compounds.

#### 2.2.4. Influence of the Selected Compounds on Metabolic Activity

When a semi-quantitative API ZYM micro-method was applied for the determination of influence of the selected compounds on activity of 19 hydrolases of *S. aureus*, it appeared that the *S. aureus* ATCC 29,213 reference strain exhibited activity of 5 enzymes, namely alkaline phosphatase, esterase (C4), esterase lipase (C8), acid phosphatase and naphthol-AS-BI-phosphohydrolase. Analysis of the enzymatic activity of bacterial cells treated with the selected agents at ½ × MIC, results of which are shown in Table 4, revealed a statistically significant decrease in the activity of alkaline phosphatase, esterase (C4) and esterase lipase (C8). The activity of two other enzymes was not affected.

In order to explain whether the observed reduction of activity of some enzymes could be a consequence of the killing of bacterial cells, the determination of CFU number in treated and control samples was performed. No difference was observed (no killing effect in the presence of the agents at a concentration of ½ MIC during 1 h incubation was observed). It clearly indicates that treatment of the cells with the selected substances (at a concentration lower than MIC) importantly affected the activity of some of the important enzymes, however without any lethal effect.

Therefore, the outcomes of our biological investigation revealed the very high antistaphylococcal activity against *S. aureus ATCC 25923*, *S. aureus ATCC 29,213* and *S. epidermidis ATCC12228* of some synthesized anthra[1,2-*d*][1,2,3]triazine-4,7,12(3*H*)-triones at the concentration of 0.125–0.5 µg/mL, while randomly selected antibiotics have MIC in the range of 0.125–8.0 µg/mL. The high antistaphylococcal potential of the most active compounds, namely **32**–**34** and **36**, has been also confirmed against clinical isolates of *S. aureus*, including the MRSA strains. The obtained results create the prospect of their further in-depth studies as effective antibacterial agents.

## 3. Materials and Methods

### 3.1. General Information

Melting points were measured in open to air glass capillaries using a B-540 melting point apparatus (Büchi, New Castle, DE, USA) and are uncorrected. Elemental analysis was performed on a 2400 CHN-analyzer (PerkinElmer, Wilmington, DE, USA) and the results were found to be in good agreement with the calculated values. ^1^H-NMR spectra (400 MHz) and ^13^C-NMR spectra (100 MHz) were recorded in DMSO-*d*_6_ (unless otherwise indicated) on a Mercury-400 spectrometer (Varian, Palo. Alto, CA, USA) with TMS as an internal standard. Mass spectra were recorded on a 1100 Series G1956B LC/MSD SL LCMS system (Agilent, Santa Clara, CA, USA) using electrospray ionization at atmospheric pressure (70 eV). X-ray structural analysis of a single crystal of compound **22** was performed at 173 K on a Smart Apex II diffractometer (Bruker, Madison, WI, USA) operating in the ω scans mode. The intensity data were collected within the range of 2.19 ≤ θ ≤ 26.72° using Mo-K_α_ radiation (λ = 0.71078 Å). The intensities of 11,753 reflections were collected (2580 unique reflections, R_merg_ = 0.0526). The structure was solved by direct methods and refined by the full-matrix least-squares technique in the anisotropic approximation for non-hydrogen atoms using the Bruker SHELXTL program package [38]. All chemicals were of reagent grade and used without further purification. The solvents were purified according to the standard procedures [39]. 1-Amino-9,10-dioxo-9,10-dihydroanthracene-2-carboxylic acid (**1**) and 1-amino-4-bromo-9,10-dioxo-9,10-dihydroanthracene-2-carboxylic acid (**2**) were purchased from Sigma-Aldrich (St. Louis, MO, USA).

### 3.2. Chemistry

*1-Amino-9,10-dioxo-9,10-dihydroanthracene-2-carboxamide* (**5**). To 1-amino-9,10-dioxo-9,10-dihydro-anthracene-2-carboxylic acid (**1**, 0.5 g, 1.87 mmol) in benzene (30 mL) pyridine (0.023 mL, 0.28 mmol) and thionyl chloride (0.41 mL, 5.61 mmol) were added and the mixture boiled for 5 h at 80 °C with a calcium chloride drying tube. The reaction mixture was cooled to room temperature. The solvent was distilled off under vacuum. The residue was added portionwise to 25% ammonia solution (50 mL) at −15 ° C and kept at this temperature for 1 h. After the mixture was gradually heated to 80 °C, kept at this temperature for 3 h, then cooled to room temperature, acidified with 10% HCl to pH = 6, filtered and washed with water. Compound **3** did not require additional purification. Yield 90%; m.p.: 299–300 °C (298–299 °C in [40]); ^1^H-NMR: *δ* = 7.31 (d, 2H, NH_2_), 7.61 (m, 1H_Ar_), 7.79–7.84 (m, 2H, NH_2_), 7.98–8.13 (m, 4H_Ar_), 9.05 (s, 1H_Ar_). ^13^C-NMR: *δ* = 113.21, 113.31, 121.77, 126.12, 126.45, 132.06, 133.45, 134.26, 134.52, 135.17, 135.68 (C_Ar_), 151.96 (C-NH_2_); 169.76, 182.43, 183.98 (C=O). LC-MS (70 eV): *m*/*z* = 267 [M + 1]^+^ (100%). Anal. Calcd. for C_15_H_10_N_2_O_3_: C, 67.67; H, 3.79; N, 10.52. Found: C, 67.75; H, 3.72; N, 10.58.

*1-Amino-4-bromo-9,10-dioxo-9,10-dihydroanthracene-2-carboxamide* (**14**). This compound was obtained similarly to **5**. Yield 89%; m.p. > 300 °C: ^1^H-NMR (CF_3_COOD): *δ* = 7.56–8.03 (m, 4H, H_Ar_+NH_2_), 8.11–8.84 (m, 5H, H_Ar_+NH_2_). ^13^C-NMR (CF_3_COOD): *δ* = 122.38, 125.82, 127.57, 127.72, 132.13, 132.88, 134.31, 135.04, 135.08, 135.67, 136.37 (C_Ar_), 142.13 (C-NH_2_); 169.92, 182.62, 185.96 (C=O). LC-MS (70 eV): *m*/*z* = 346 [M + 1]^+^ (100%). Anal. Calcd. for C_15_H_9_BrN_2_O_3_: C, 52.20; H, 2.63; N, 8.12. Found: C, 52.26; H, 2.69; N, 8.17.

#### 3.2.1. General Procedure for the Synthesis of Amides 6–13, 15–21

To the corresponding amino acid **1** or **2** (1.87 mmol) in benzene (30 mL), pyridine (0.023 mL, 0.28 mmol) and thionyl chloride (0.41 mL, 5.61 mmol) were added. The mixture was boiled for 5 h at 80 °C with a calcium chloride drying tube, and then the solvent was distilled off under vacuum. Benzene (30 mL) was added to the resulting residue at room temperature, then the corresponding aniline (2.06 mmol) and triethylamine (0.29 mL, 2.06 mmol) were added (in the case of cyclohexylamine, *N*,*N*-diisopropylethylamine was used). The mixture was boiled at 80 °C for 12 h, cooled to room temperature and the solvent was distilled off under vacuum. The resulting precipitate was suspended in water (100 mL), filtered, washed with water (150 mL and dried. If necessary, amides can be recrystallized from acetic acid.

*1-Amino-9,10-dioxo-N-(2-(trifluoromethyl)phenyl)-9,10-dihydroanthracene-2-carboxamide* (**6**). Yield 89%; m.p.: 180–182 °C (sublimation); ^1^H-NMR: *δ* = 7.49 (d, *J* = 7.8 Hz, 1H_Ar_), 7.59 (t, *J* = 8.3 Hz, 2H_Ar_), 7.74–7.93 (m, 5H_Ar_), 8.12 (d, *J* = 7.3 Hz, 2H_Ar_), 8.20 (d, *J* = 7.6 Hz, 1H_Ar_), 8.77 (s, 1H_Ar_), 10.42 (br.s, 1H, NH). ^13^C-NMR: *δ* = 114.01, 119.68 (q, *J* = 32 Hz, C-CF_3_), 122.65, 125.55 (q, *J* = 268 Hz, CF_3_), 126.75, 127.06, 128.32, 131.84, 132.64, 133.70, 134.15, 134.76, 135.17, 135.67, 136.54, 151.94 (C_Ar_); 167.91, 182.98, 184.66 (C=O). LC-MS (70 eV): *m*/*z* = 411 [M + 1]^+^ (100%). Anal. Calcd. for C_22_H_13_F_3_N_2_O_3_: C, 64.39; H, 3.19; N, 6.83. Found: C, 64.39; H, 3.15; N, 6.87.

*1-Amino-9,10-dioxo-N-(m-tolyl)-9,10-dihydroanthracene-2-carboxamide* (**7**). Yield 91%; m.p.: 225–226 °C; ^1^H-NMR: *δ* = 2.31 (s, 3H, CH_3_), 6.94 (d, *J* = 6.2 Hz, 1H_Ar_), 7.23 (t, *J* = 7.0 Hz, 1H_Ar_), 7.43–7.63 (m, 5H, H_Ar_+NH_2_), 7.86 (dd, *J* = 22.9, 6.0 Hz, 2H_Ar_), 8.02-8.22 (m, 3H_Ar_), 10.39 (br s, 1H, NH). ^13^C-NMR: δ = 21.68 (CH_3_), 113.75, 114.14, 118.34, 121.69, 125.30, 126.74, 127.08, 128.95, 132.67, 134.11, 134.80, 135.14, 135.69, 136.13, 138.31, 139.01 (C_Ar_), 166.54, 182.98, 184.63 (C=O). LC-MS (70 eV): *m*/*z* = 357 [M + 1]^+^ (100%). Anal. Calcd. for C_22_H_16_N_2_O_3_: C, 74.15; H, 4.53; N, 7.86. Found: C, 74.09; H, 4.51; N, 7.91.

*1-Amino-N-cyclohexyl-9,10-dioxo-9,10-dihydroanthracene-2-carboxamide* (**8**). Yield 91%; m.p.: 289-290 °C (289–290 °C in [40]); ^1^H-NMR: δ = 1.29 (m, 6H, CH_2_), 1.59-1.91 (m, 4H, CH_2_), 3.76 (s, 1H, CH), 7.36 (s, 3H_Ar__,_ CH+NH_2_), 7.89 (m, 3H_Ar_), 8.15 (m, 3H_Ar__,_ CH+NH). ^13^C-NMR: δ = 25.03, 30.79, 32.66 (CH_2_), 48.76 (CH), 114.16, 127.07, 128.80, 132.69, 134.12, 135.17, 135.75, 135.82, 151.74 (C_Ar_), 166.91, 183.07, 184.59 (C=O). LC-MS (70 eV): *m*/*z* = 349 [M + 1]^+^ (100%). Anal. Calcd. for C_21_H_20_N_2_O_3_: C, 72.40; H, 5.79; N, 8.04. Found: C, 72.45; H, 5.75; N, 8.01.

*1-Amino-N-(3-fluorophenyl)-9,10-dioxo-9,10-dihydroanthracene-2-carboxamide* (**9**). Yield 90%; m.p.: 240–242 °C; ^1^H-NMR: δ = 6.95 (t, *J* = 7.6 Hz, 1H_Ar_), 7.32–7.54 (m, 4H_,_ H_Ar_+NH_2_), 7.69 (d, *J* = 11.4 Hz, 1H_Ar_), 7.80–7.93 (m, 3H_Ar_), 8.07 (dd, *J* = 21.4, 6.6 Hz, 2H_Ar_), 8.15–8.20 (m, 1H_Ar_), 10.63 (s, 1H, NH). ^13^C-NMR: δ = 113.83, 114.07, 116.78, 124.05, 126.74, 127.08, 130.70, 130.77, 132.64, 134.15, 134.76, 135.17, 135.80, 136.32, 140.81, 151.59 (C_Ar_), 162.49 (d, *J* = 241.1 Hz, C-F), 166.78, 182.96, 184.65 (C=O). LC-MS (70 eV): *m*/*z* = 361 [M + 1]^+^ (100%). Anal. Calcd. for C_21_H_13_FN_2_O_3_: C, 70.00; H, 3.64; N, 7.77. Found: C, 70.03; H, 3.60; N, 7.74.

*1-Amino-N-(4-fluorophenyl)-9,10-dioxo-9,10-dihydroanthracene-2-carboxamide* (**10**). Yield 88%; m.p.: 262–264 °C; ^1^H-NMR: *δ* = 7.19 (s, 2H_Ar_), 7.32–7.44 (m, 2H, NH_2_), 7.73 (s, 2H_Ar_), 7.83 (d, *J* = 28.3 Hz, 3H_Ar_), 8.01–8.09 (m, 2H), 8.13–8.17 (m, 1H_Ar_), 10.50 (br s, 1H, NH). ^13^C-NMR: δ = 116.08, 117.89, 124.37, 125.17, 125.50, 126.27, 129.05, 130.95, 134.78, 136.28, 136.92, 137.30, 137.72, 138.34, 153.78 (C_Ar_), 161.19 (d, *J* = 241.4, C-F), 168.64, 185.11, 186.77 (C=O). LC-MS (70 eV): *m*/*z* = 361 [M + 1]^+^ (100%). Anal. Calcd. for C_21_H_13_FN_2_O_3_: C, 70.00; H, 3.64; N, 7.77. Found: C, 69.98; H, 3.61; N, 7.79.

*1-Amino-N-(3-chlorophenyl)-9,10-dioxo-9,10-dihydroanthracene-2-carboxamide* (**11**). Yield 87%; m.p.: 252–253 °C; ^1^H-NMR (CF_3_COOD): δ = 7.39–7.45 (m, 4H_Ar_), 7.67 (s, 1H), 8.05 (m, 2H_Ar_), 8.44 (d, *J* = 11.0 Hz, 4H_Ar_), 8.56 (d, *J* = 7.4 Hz, 1H_Ar_), 11.52 (s, 1H, NH). ^13^C-NMR (CF_3_COOD): δ = 116.95, 123.90, 125.89, 128.14, 130.79, 131.25, 132.20, 133.49, 135.10, 135.27, 135.91, 137.39, 138.17, 139.03, 139.62, 140.06 (C_Ar_), 168.73, 185.40, 189.75 (C=O). LC-MS (70 eV): *m*/*z* = 377 [M + 1]^+^ (100%). Anal. Calcd. for C_21_H_13_ClN_2_O_3_: C, 66.94; H, 3.48; N, 7.43. Found: C, 66.99; H, 3.43; N, 7.45.

*1-Amino-N-(4-chlorophenyl)-9,10-dioxo-9,10-dihydroanthracene-2-carboxamide* (**12**). Yield 89%; m.p.: 265–266 °C; ^1^H-NMR (CF_3_COOD): δ = 7.38–7.45 (m, 4H_,_ H_Ar_+NH_2_), 7.97 (m, 3H_Ar_), 8.33–8.39 (m, 4H_Ar_), 8.70 (s, 1H_Ar_), 11.51 (s, NH). ^13^C-NMR (CF_3_COOD): δ = 123.77, 124.02, 125.43, 128.38, 129.52, 129.67, 130.43, 131.52, 132.04, 132.84, 132.91, 133.07, 133.96, 134.46, 136.29, 136.57, 136.89 (C_Ar_), 165.69, 182.76, 186.67 (C=O). LC-MS (70 eV): *m*/*z* = 377 [M + 1]^+^ (100%). Anal. Calcd. for C_21_H_13_ClN_2_O_3_: C, 66.94; H, 3.48; N, 7.43. Found: C, 66.93; H, 3.50; N, 7.41.

*1-Amino-N-(2-methoxyphenyl)-9,10-dioxo-9,10-dihydroanthracene-2-carboxamide* (**13**). Yield 89%; m.p.: 187–189 °C [41]; ^1^H-NMR (CF_3_COOD): δ = 3.90 (s, 3H, OCH_3_), 7.03 (t, *J* = 7.4 Hz, 2H_Ar_), 7.30 (t, *J* = 8.3 Hz, 1H_Ar_), 7.77 (d, *J* = 7.6 Hz, 1H_Ar_), 7.97 (m, 3H_Ar_), 8.38 (m, 3H_Ar_), 8.47 (d, *J* = 8.2 Hz, 1H_Ar_), 8.71 (d, *J* = 8.2 Hz, 1H_Ar_), 11.52 (s, NH). ^13^C-NMR (CF_3_COOD): δ = 55.09 (CH_3_), 111.43, 121.19, 123.10, 123.37, 127.85, 128.29, 128.86, 129.71, 131.28, 131.93, 132.81, 133.17, 134.20, 136.22, 136.49 (C_Ar_), 151.10, 182.68, 186.54 (C=O). LC-MS (70 eV): *m*/*z* = 373 [M + 1]^+^ (100%). Anal. Calcd. for C_22_H_16_N_2_O_4_: C, 70.96; H, 4.33; N, 7.52. Found: C, 70.92; H, 4.30; N, 7.56.

*1-Amino-4-bromo-N-(3-fluorophenyl)-9,10-dioxo-9,10-dihydroanthracene-2-carboxamide* (**15**). Yield 90%; m.p.: 253–255 °C; ^1^H-NMR: δ = 6.95 (s, 1H_Ar_), 7.34–7.47 (m, 4H, NH_2_+H_Ar_), 7.77–7.84 (m, 3H_Ar_), 8.04 (d, *J* = 20.7 Hz, 2H), 8.15 (m, 1H_Ar_), 10.72 (s, 1H, NH). ^13^C-NMR: δ = 126.53, 126.77, 130.76, 133.18, 133.47, 133.73, 134.22, 134.73, 141.77, 151.05 (C_Ar_), 162.44 (d, *J* = 241.0 Hz, C-F), 165.25, 182.37, 184.04 (C=O). LC-MS (70 eV): *m*/*z* = 440 [M + 1]^+^ (100%). Anal. Calcd. for C_21_H_12_BrFN_2_O_3_: C, 57.42; H, 2.75; N, 6.38. Found: C, 57.47; H, 2.71; N, 6.43.

*1-Amino-4-bromo-N-(2-methoxyphenyl)-9,10-dioxo-9,10-dihydroanthracene-2-carboxamide* (**16**). Yield 91%; m.p.: 225–226 °C; ^1^H-NMR: δ = 3.83 (s, 3H, CH_3_), 6.95-7.38 (m, 4H_Ar_), 7.52–7.66 (m, 1H_Ar_), 7.79–7.93 (m, 2H, NH_2_), 8.06-8.24 (m, 3H_Ar_), 8.84 (m, 1H), 10.03 (s, 1H, NH). ^13^C-NMR: δ = 56.20 (CH_3_), 106.33, 112.19, 120.63, 126.23, 126.45, 126.61, 126.84, 127.34, 128.80, 133.32, 133.41, 133.87, 134.31, 134.83, 141.82, 151.18, 153.00 (C_Ar_), 161.38, 182.57, 184.15 (C=O). LC-MS (70 eV): *m*/*z* = 452 [M + 1]^+^ (100%). Anal. Calcd. for C_22_H_15_BrN_2_O_4_: C, 58.55; H, 3.35; N, 6.21. Found: C, 58.59; H, 3.31; N, 6.24.

*1-Amino-4-bromo-N-cyclohexyl-9,10-dioxo-9,10-dihydroanthracene-2-carboxamide* (**17**). Yield 89%; m.p.: >300 °C (298–299 °C in [40]); ^1^H-NMR: δ = 1.29 (m, 5H, CH_2_), 1.78 (m, 5H, CH_2_), 3.73 (m, 1H, CH), 7.35 (s, 1H_Ar_), 7.81–7.88 (s, 2H, NH_2_), 7.99–8.13 (m, 4H_Ar_), 8.65 (s, 1H_Ar_). ^13^C-NMR: δ = 25.32, 25.68, 32.58 (CH_2_), 48.90 (CH), 124.81, 126.52, 126.59, 126.80, 128.81, 129.22, 133.02, 133.25, 133.83, 134.21, 134.76, 141.22 (C_Ar_), 165.46, 182.50, 182.63 (C=O). LC-MS (70 eV): *m*/*z* = 428 [M + 1]^+^ (100%). Anal. Calcd. for C_21_H_19_BrN_2_O_3_: C, 59.03; H, 4.48; N, 6.56. Found: C, 59.00; H, 4.51; N, 6.59.

*1-Amino-4-bromo-N-(4-fluorophenyl)-9,10-dioxo-9,10-dihydroanthracene-2-carboxamide* (**18**). Yield 87%; m.p.: 273–275 °C; ^1^H-NMR: *δ* = 7.19 (s, 2H_Ar_), 7.71 (s, 2H, NH_2_), 7.81 (s, 3H_Ar_), 8.05 (d, *J* = 22.5 Hz, 3H_Ar_), 8.15 (s, 1H_Ar_), 10.61 (s, 1H, NH). ^13^C-NMR: δ = 106.32, 115.82, 123.09, 123.26, 124.84, 126.53, 126.77, 133.20, 133.75, 134.22, 134.73, 141.68, 150.98 (C_Ar_), 159.15 (d, *J* = 241.1 Hz, C-F), 164.97, 182.39, 184.04 (C=O). LC-MS (70 eV): *m*/*z* = 440 [M + 1]^+^ (100%). Anal. Calcd. for C_21_H_12_BrFN_2_O_3_: C, 57.42; H, 2.75; N, 6.38. Found: C, 57.46; H, 2.71; N, 6.43.

*1-Amino-4-bromo-N-(3-chlorophenyl)-9,10-dioxo-9,10-dihydroanthracene-2-carboxamide* (**19**). Yield 90%; m.p.: 243–245 °C; ^1^H-NMR (CF_3_COOD): δ = 7.21–7.44 (m, 4H, H_Ar_+NH_2_), 7.80–8.05 (m, 4H_Ar_), 8.21–8.39 (m, 3H_Ar_), 11.51 (1H, NH). ^13^C-NMR (CF_3_COOD): δ = 119.06, 119.96, 120.91, 122.20, 124.11, 126.23, 126.70, 127.14, 129.32, 130.63, 132.35, 133.91, 135.05, 137.34, 138.29, 141.87, 150.38 (C_Ar_), 166.14, 182.88, 184.50 (C=O). LC-MS (70 eV): *m*/*z* = 456 [M + 1]^+^ (100%). Anal. Calcd. for C_21_H_12_BrClN_2_O_3_: C, 55.35; H, 2.65; N, 6.15. Found: C, 55.39; H, 2.61; N, 6.18.

*1-Amino-4-bromo-N-(4-chlorophenyl)-9,10-dioxo-9,10-dihydroanthracene-2-carboxamide* (**20**). Yield 87%; m.p.: 255–256 °C; ^1^H-NMR: δ = 7.44 (d, *J* = 8.6 Hz, 2H_Ar_), 7.76 (d, *J* = 8.6 Hz, 2H_Ar_), 7.85–7.92 (m, 4H_Ar_), 8.10 (d, *J* = 7.2 Hz, 1H_Ar_), 8.17 (d, *J* = 7.5 Hz, 1H_Ar_), 8.20 (s, 1H_Ar_), 10.74 (s, 1H, NH). ^13^C-NMR: δ = 115.14, 122.27, 124.52, 126.08, 126.31, 127.94, 128.55, 132.76, 132.97, 133.30, 133.79, 137.34, 141.25, 150.48 (C_Ar_), 164.60, 181.99, 183.64 (C=O). LC-MS (70 eV): *m*/*z* = 456 [M + 1]^+^ (100%). Anal. Calcd. for C_21_H_12_BrClN_2_O_3_: C, 55.35; H, 2.65; N, 6.15. Found: C, 55.33; H, 2.62; N, 6.13.

*1-Amino-4-bromo-9,10-dioxo-N-(2-(trifluoromethyl)phenyl)-9,10-dihydroanthracene-2-carboxamide* (**21**). Yield 89%; m.p.: 285–286 °C (sublimation); ^1^H-NMR: *δ* = 7.56–7.66 (m, 2H_Ar_), 7.76 (d, *J* = 7.6 Hz, 1H_Ar_), 7.82 (d, *J* = 8.0 Hz, 1H_Ar_), 7.88-7.91 (m, 2H_Ar_), 8.13 (d, *J* = 7.5 Hz, 2H_Ar_), 8.20 (d, *J* = 7.7, 1H_Ar_), 8.28 (s, 1H_Ar_), 8.84 (s, 1H_Ar_), 10.48 (s, 1H, NH). ^13^C-NMR: *δ* = 116.12, 119.76 (q, *J* = 35.2 Hz, C-CF_3_), 123.86, 124.55 (q, *J* = 268 Hz, CF_3_), 126.61, 126.82, 128.35, 131.61, 133.41, 133.63, 133.95, 134.28, 134.77, 141.82, 151.38 (C_Ar_), 166.55, 182.52, 184.22 (C=O). LC-MS (70 eV): *m*/*z* = 490 [M + 1]^+^ (100%). Anal. Calcd. for C_22_H_12_BrF_3_N_2_O_3_: C, 54.01; H, 2.47; N, 5.73. Found: C, 54.04; H, 2.43; N, 5.76.

#### 3.2.2. General Procedure for the Synthesis of Anthra[1,2-*d*][1,2,3]triazine-4,7,12(3*H*)-triones 22–38

To the corresponding amide **5**–**13** (1.88 mmol) in glacial acetic acid (50 mL) (in the case of amides **14**–**21** 100–150 mL of glacial acetic acid was used), a first portion of NaNO_2_ (0.168 g, 2.44 mmol) in water (1.5 mL) was added with the stirring at room temperature, After 3 h, the same amount of NaNO_2_ in water (1.5 mL) was added. The mixture was stirred for 9 h at room temperature and then filtered. The filtrate was poured into water (200 mL, in the case of amides **14**–**21** 600 mL of water), acidified with a 10% HCl solution to pH = 6. The resulting precipitate was filtered off, washed with water and dried in air.

*Anthra[1,2-d][1,2,3]triazine-4,7,12(3H)-trione* (**22**). Yield 90%; m.p.: 140-142 °C (decomposition); ^1^H-NMR: *δ* = 7.88–7.91 (m, 2H_Ar_), 8.10 (m, 2H_Ar_), 8.49–8.33 (m, 2H_Ar_), 15.38 (1H, br s, NH). ^13^C-NMR: *δ* = 124.43, 126.82, 127.17, 129.57, 130.39, 132.19, 134.58, 135.17, 135.53, 139.03, 141.94 (C_Ar_); 155.22, 182.04, 182.28 (C=O). LC-MS (70 eV): *m*/*z* = 278 [M + 1]^+^ (100%). Anal. Calcd. for C_15_H_7_N_3_O_3_: C, 64.99; H, 2.55; N, 15.16. Found: C, 65.01; H, 2.51; N, 15.19.

*Crystallography: X-ray structure determination of compound***22**. Crystal data for **22**: C_15_H_7_N_3_O_4_ ˣH_2_O, M = 295.25, monoclinic, space group P2_1_/c, *a* = 7.070(3), *b* = 12.687(5), *c* = 13.816(5) Å, *β* = 95.842(11)°, V = 1232.9(8)Å^3^, Z = 4, d_c_ = 1.591 g·cm^−3^, μ = 0.119 mm^−1^, F(000) = 608, crystal size ca. 0.09 × 0.11 × 0.50 mm. All CH hydrogen atoms were placed at calculated positions and refined as ‘riding’ model and NH hydrogen atom was located at DF synthesis and refined isotropically. Convergence was obtained at R1 = 0.0613 and wR2 = 0.1134 for 1319 observed reflections with I ≥ 2σ(I), R1 = 0.1465 and wR2 = 0.1474, GOF = 1.039 for 2580 independent reflections, 211 parameters, the largest and minimal peaks in the final difference map 0.18 and −0.18 e/Å^3^. Full crystallographic details have been deposited at Cambridge Crystallographic Data Centre (CCDC). Any request to the CCDC for these materials should quote the full literature citation and reference number CCDC 1966785.

*3-(2-(Trifluoromethyl)phenyl)anthra[1,2-d][1,2,3]triazine-4,7,12(3H)-trione* (**23**). Yield 72%; m.p.: 225–226 °C (decomposition); ^1^H-NMR (400 MHz, DMSO-*d_6_*): *δ* = 7.89 (t, *J* = 7.5 Hz, 1H_Ar_), 7.94–8.01 (m, 4H_Ar_), 8.06 (d, *J* = 7.8 Hz, 1H_Ar_), 8.20 (d, *J* = 7.4 Hz, 2H_Ar_), 8.69 (d, *J* = 8.2 Hz, 1H_Ar_), 8.74 (d, J = 8.2 Hz, 1H_Ar_). ^13^C-NMR (100 MHz, DMSO-*d_6_*): *δ* = 119.01 (q, *J* = 32 Hz, C-CF_3_), 122.44, 123.81, 124.46, 125.03 (q, *J* = 273 Hz, CF_3_), 126.42, 126.91, 127.27, 127.98, 129.84, 130.99, 131.70, 132.38, 134.73, 135.26, 135.56, 136.33, 139.54, 140.84 (C_Ar_); 154.80, 182.00, 182.14 (C=O). LC-MS (70 eV): *m*/*z* = 422 [M + 1]^+^ (100%). Anal. Calcd. for C_22_H_10_F_3_N_3_O_3_: C, 62.72; H, 2.39; N, 9.97. Found: C, 62.67; H, 2.43; N, 10.01.

*3-(m-Tolyl)anthra[1,2-d][1,2,3]triazine-4,7,12(3H)-trione* (**24**). Yield 73%; m.p.: 230-231 °C (decomposition); ^1^H-NMR: *δ* = 1.90 (s, 3H, CH_3_), 7.39-7.60 (m, 3H_Ar_), 7.88–8.03 (m, 3H_Ar_), 8.20 (d, *J* = 8.4 Hz, 2H_Ar_), 8.59-8.76 (m, 2H_Ar_). ^13^C-NMR: *δ* = 124.02, 124.41, 126.90, 127.25, 127.30, 129.32, 129.40, 130.33, 130.97, 132.35, 134.68, 135.13, 135.19, 135.57, 138.92, 139.09, 140.87 (C_Ar_); 154.41, 182.04, 182.26 (C=O). LC-MS (70 eV): *m*/*z* = 368 [M + 1]^+^ (100%). Anal. Calcd. for C_22_H_13_N_3_O_3_: C, 71.93; H, 3.57; N, 11.44. Found: C, 71.97; H, 3.52; N, 11.46.

*3-Cyclohexylanthra[1,2-d][1,2,3]triazine-4,7,12(3H)-trione* (**25**). Yield 71%; m.p.: 215 °C (decomposition); ^1^H-NMR: *δ* = 1.54 (dd, *J* = 36.6, 9.0 Hz, 4H, CH_2_), 1.88-2.08 (m, 6H, CH_2_), 4.82-4.95 (m, 1H, CH), 7.86–7.97 (m, 2H_Ar_), 8.12 (s, 2H_Ar_), 8.45–8.60 (m, 2H_Ar_). ^13^C-NMR: *δ* = 25.45, 25.71, 31.81 (CH_2_), 57.02 (C-H), 123.12, 126.84, 127.25, 129.00, 129.73, 130.88, 131.93, 133.44, 134.61, 135.54, 138.90, 140.94 (C_Ar_); 153.92, 181.97, 182.28 (C=O). LC-MS (70 eV): *m*/*z* = 360 [M + 1]^+^ (100%). Anal. Calcd. for C_21_H_17_N_3_O_3_: C, 70.18; H, 4.77; N, 11.69. Found: C, 70.21; H, 4.74; N, 11.72.

*3-(3-Fluorophenyl)anthra[1,2-d][1,2,3]triazine-4,7,12(3H)-trione* (**26**). Yield 69%; m.p.: 225 °C (decomposition); ^1^H-NMR: δ = 7.39–7.49 (m, 1H_Ar_), 7.58–7.73 (m, 3H_Ar_), 7.91–7.99 (m, 2H_Ar_), 8.15–8.23 (m, 2H_Ar_), 8.59–8.74 (m, 2H_Ar_). ^13^C-NMR: δ = 116.61, 116.78, 123.21, 124.40, 126.93, 127.27, 127.32, 129.46, 130.50, 130.96, 131.29, 132.40, 134.70, 135.22, 135.57, 140.74 (C_Ar_), 162.12 (d, *J* = 246.0 Hz, C-F), 154.39, 182.03, 182.22 (C=O). LC-MS (70 eV): *m*/*z* = 372 [M + 1]^+^ (100%). Anal. Calcd. for C_21_H_10_FN_3_O_3_: C, 67.93; H, 2.71; N, 11.32. Found: C, 67.96; H, 2.68; N, 11.36.

*3-(4-Fluorophenyl)anthra[1,2-d][1,2,3]triazine-4,7,12(3H)-trione* (**27**). Yield 75%; m.p.: 230–232 °C (decomposition); ^1^H-NMR: *δ* = 7.42–7.56 (m, 2H_Ar_), 7.74–7.85 (m, 2H_Ar_), 7.89–8.03 (m, 3H), 8.11–8.25 (m, 2H), 8.66 (d, *J* = 30.3 Hz, 1H). ^13^C-NMR (100 MHz, CF_3_COOD): δ = 111.43, 116.21, 124.69, 128.37, 128.58, 130.15, 131.54, 131.78, 134.51, 134.69, 136.54, 137.13, 140.00, 142.25 (C_Ar_), 162.05 (d, *J* = 248.6 Hz, C-F), 160.19, 178.17, 178.75 (C=O). LC-MS (70 eV): *m*/*z* = 372 [M + 1]^+^ (100%). Anal. Calcd. for C_21_H_10_FN_3_O_3_: C, 67.93; H, 2.71; N, 11.32. Found: C, 67.89; H, 2.73; N, 11.29.

*3-(3-Chlorophenyl)anthra[1,2-d][1,2,3]triazine-4,7,12(3H)-trione* (**28**). Yield 69%; m.p.: 233 °C (decomposition); ^1^H-NMR (CF_3_COOD): δ = 7.29 (d, *J* = 7.6 Hz, 1H_Ar_), 7.35 (t, *J* = 8.0 Hz, 1H_Ar_), 7.41 (d, *J* = 7.8 Hz, 1H_Ar_), 7.64 (s, 1H_Ar_), 8.00–8.03 (m, 2H_Ar_), 8.44 (dd, *J* = 11.4 Hz, 8.7, 2H_Ar_), 8.85 (d, *J* = 8.2 Hz, 1H_Ar_), 9.23 (d, *J* = 8.2 Hz, 1H_Ar_). ^13^C-NMR (CF_3_COOD): δ = 111.50, 120.29, 122.43, 128.04, 128.45, 128.66, 130.39, 131.60, 131.86, 134.51, 134.78, 135.52, 136.62, 137.19, 140.08, 142.35 (C_Ar_), 160.02, 178.23, 178.80 (C=O). LC-MS (70 eV): *m*/*z* = 388 [M + 1]^+^ (100%). Anal. Calcd. for C_21_H_10_ClN_3_O_3_: C, 65.05; H, 2.60; N, 10.84. Found: C, 65.08; H, 2.56; N, 10.81.

*3-(4-Chlorophenyl)anthra[1,2-d][1,2,3]triazine-4,7,12(3H)-trione* (**29**). Yield 67%; m.p.: 251 °C (decomposition); ^1^H-NMR (CF_3_COOD): δ = 7.39 (d, *J* = 7.3 Hz, 2H_Ar_), 7.52 (d, *J* = 7.0 Hz, 2H_Ar_), 8.00–8.05 (m, 2H_Ar_), 8.41–8.47 (m, 2H_Ar_), 8.82–8.90 (m, 1H_Ar_), 9.20–9.27 (m, 1H_Ar_). ^13^C-NMR (CF_3_COOD): δ = 111.42, 123.52, 128.38, 128.59, 129.47, 131.55, 131.78, 132.89, 134.51, 134.71, 136.55, 137.09, 137.17, 140.02, 142.34 (C_Ar_), 159.89, 178.17, 178.76 (C=O). LC-MS (70 eV): *m*/*z* = 388 [M + 1]^+^ (100%). Anal. Calcd. for C_21_H_10_ClN_3_O_3_: C, 65.05; H, 2.60; N, 10.84. Found: C, 65.02; H, 2.58; N, 10.86.

*3-(2-Methoxyphenyl)anthra[1,2-d][1,2,3]triazine-4,7,12(3H)-trione* (**30**). Yield 78%; m.p.: 216 °C (decomposition); ^1^H-NMR (CF_3_COOD): δ = 3.90 (s, 3H, CH_3_), 7.06 (d, *J* = 8.1 Hz, 1H_Ar_), 7.33 (t, *J* = 7.8 Hz, 1H_Ar_), 7.80 (d, *J* = 7.8 Hz, 1H_Ar_), 8.00–8.03 (m, 3H_Ar_), 8.44 (dd, *J* = 10.8 Hz, 8.9 Hz, 2H_Ar_), 8.82 (d, *J* = 8.1 Hz, 1H_Ar_), 9.24 (d, *J* = 8.2 Hz, 1H_Ar_). ^13^C-NMR (CF_3_COOD): *δ* = 55.18 (CH_3_), 111.51, 121.27, 123.01, 123.48, 128.64, 131.58, 131.85, 134.44, 134.73, 136.60, 137.11, 137.23, 140.13, 142.59 (C_Ar_);, 151.26 (C-OMe), 159.80, 178.24, 178.81 (C=O). LC-MS (70 eV): *m*/*z* = 384 [M + 1]^+^ (100%). Anal. Calcd. for C_22_H_13_N_3_O_4_: C, 68.93; H, 3.42; N, 10.96. Found: C, 68.95; H, 3.39; N, 10.99.

*6-Bromoanthra[1,2-d][1,2,3]triazine-4,7,12(3H)-trione* (**31**). Yield 78%; m.p.: 155 °C (decomposition); ^1^H-NMR: *δ* = 7.92 (d, *J* = 3.4 Hz, 2H_Ar_), 8.08 (d, *J* = 8.8 Hz, 2H_Ar_), 8.67 (s, 1H_Ar_), 15.45 (br s, 1H, NH). ^13^C-NMR: *δ* = 123.54, 124.15, 126.32, 126.93, 133.50, 134.41, 134.67, 135.00, 135.72, 141.04 (C_Ar_); 153.96, 181.71, 181.94 (C=O). LC-MS (70 eV): *m*/*z* = 357 [M + 1]^+^ (100%). Anal. Calcd. for C_15_H_6_BrN_3_O_3_: C, 50.59; H, 1.70; N, 11.80. Found: C, 50.62; H, 1.67; N, 11.83.

*6-Bromo-3-(3-fluorophenyl)anthra[1,2-d][1,2,3]triazine-4,7,12(3H)-trione* (**32**). Yield 65%; m.p.: 235–237 °C (decomposition); ^1^H-NMR: δ = 7.37–7.52 (m, 1H_Ar_), 7.65 (m, 2H_Ar_), 7.87–8.02 (m, 2H_Ar_), 8.08–8.30 (m, 3H_Ar_), 8.62–8.91 (m, 1H_Ar_). ^13^C-NMR (CF_3_COOD): δ = 109.25, 109.46, 109.81, 114.55, 114.73, 117.37, 128.11, 128.52, 130.73, 132.79, 134.91, 135.71, 135.99, 137.69, 139.56, 140.02, 141.82 (C_Ar_), 163.11 (d, *J* = 246.5 Hz, C-F), 159.03, 177.56, 178.28 (C=O). LC-MS (70 eV): *m*/*z* = 451 [M + 1]^+^ (100%). Anal. Calcd. for C_21_H_9_BrFN_3_O_3_: C, 56.02; H, 2.01; N, 9.33. Found: C, 56.05; H, 1.99; N, 9.36.

*6-Bromo-3-(2-methoxyphenyl)anthra[1,2-d][1,2,3]triazine-4,7,12(3H)-trione* (**33**). Yield 67%; m.p.: 240 °C (decomposition); ^1^H-NMR: *δ* = 3.80 (s, 3H, CH_3_), 7.17–7.34 (m, 2H_Ar_), 7.56–7.64 (m, 2H_Ar_), 7.95 (s, 2H_Ar_), 8.11–8.20 (m, 2H_Ar_), 8.80 (s, 1H_Ar_). ^13^C-NMR: *δ* = 56.44 (CH_3_), 113.14, 121.25, 123.51, 124.66, 126.42, 127.01, 127.31, 129.40, 132.12, 133.69, 134.46, 134.76, 135.03, 136.90, 140.07 (C_Ar_); 152.80 (C-OMe), 154.86, 181.68, 181.89 (C=O). LC-MS (70 eV): *m*/*z* = 463 [M + 1]^+^ (100%). Anal. Calcd. for C_22_H_12_BrN_3_O_4_: C, 57.16; H, 2.62; N, 9.09. Found: C, 57.12; H, 2.66; N, 9.05.

*6-Bromo-3-cyclohexylanthra[1,2-d][1,2,3]triazine-4,7,12(3H)-trione* (**34**). Yield 65%; m.p.: 242–243 °C (decomposition); ^1^H-NMR: *δ* = 1.20–1.54 (m, 6H, CH_2_), 1.69–1.95 (m, 4H, CH_2_), 4.82–4.93 (m, 1H_,_ CH), 7.92 (s, 2H_Ar_), 8.08 (s, 2H_Ar_), 8.66 (s, 1H_Ar_). ^13^C-NMR: *δ* = 25.40, 25.66, 31.71 (CH_2_), 57.22 (CH), 122.76, 123.65, 126.31, 126.91, 132.99, 133.40, 134.26, 134.69, 134.99, 136.37, 139.94 (C_Ar_); 152.58, 181.58, 181.71 (C=O). LC-MS (70 eV): *m*/*z* = 439 [M + 1]^+^ (100%). Anal. Calcd. for C_21_H_16_BrN_3_O_3_: C, 57.55; H, 3.68; N, 9.59. Found: C, 57.58; H, 3.64; N, 9.62.

*6-Bromo-3-(4-fluorophenyl)anthra[1,2-d][1,2,3]triazine-4,7,12(3H)-trione* (**35**). Yield 67%; m.p.: 223 °C (decomposition); ^1^H-NMR: *δ* = 7.48 (dd, *J* = 9.2 Hz, 4.7 Hz, 2H_Ar_), 7.72–7.79 (m, 2H_Ar_), 7.90–7.98 (m, 2H_Ar_), 8.11–8.18 (m, 2H_Ar_), 8.78 (s, 1H_Ar_). ^13^C-NMR: δ = 109.84, 116.16, 116.35, 124.36, 124.43, 128.15, 128.55, 130.18, 130.32, 132.84, 134.97, 137.55, 137.71, 139.52, 140.04, 141.91 (C_Ar_), 162.08 (d, *J* = 248.7 Hz, C-F), 159.32, 177.61, 178.30 (C=O). LC-MS (70 eV): *m*/*z* = 451 [M + 1]^+^ (100%). Anal. Calcd. for C_21_H_9_BrFN_3_O_3_: C, 56.02; H, 2.01; N, 9.33. Found: C, 56.05; H, 1.97; N, 9.36.

*6-Bromo-3-(3-chlorophenyl)anthra[1,2-d][1,2,3]triazine-4,7,12(3H)-trione* (**36**). Yield 65%; m.p.: 211 °C (decomposition); ^1^H-NMR (CF_3_COOD): δ = 7.27 (d, *J* = 7.8 Hz, 1H_Ar_), 7.33 (t, *J* = 7.9 Hz, 1H_Ar_), 7.39 (d, *J* = 7.6 Hz, 1H_Ar_), 7.63 (s, 1H_Ar_), 7.98 (m, 2H_Ar_), 8.35–8.40 (m, 2H_Ar_), 9.02 (s, 1H_Ar_). ^13^C-NMR (CF_3_COOD): δ = 119.98, 122.10, 127.93, 128.18, 128.56, 130.34, 130.37, 135.48, 135.60, 136.06, 137.58, 137.73, 140.05, 141.90 (C_Ar_), 159.04, 177.61, 178.24 (C=O). LC-MS (70 eV): *m*/*z* = 467 [M + 1]^+^ (100%). Anal. Calcd. for C_21_H_9_BrClN_3_O_3_: C, 54.05; H, 1.94; N, 9.00. Found: C, 54.08; H, 1.98; N, 9.03.

*6-Bromo-3-(4-chlorophenyl)anthra[1,2-d][1,2,3]triazine-4,7,12(3H)-trione* (**37**). Yield 63%; m.p.: 223 °C (decomposition); ^1^H-NMR (CF_3_COOD): δ = 7.40 (d, *J* = 8.4 Hz, 2H_Ar_), 7.53 (d, *J* = 8.5 Hz, 2H_Ar_), 8.00 (d, *J* = 6.9 Hz, 1H_Ar_), 8.04 (d, *J* = 7.4 Hz, 1H_Ar_), 8.41 (d, *J* = 7.8 Hz, 2H_Ar_), 9.05 (s, 1H_Ar_). ^13^C-NMR (CF_3_COOD): δ = 123.20, 128.14, 128.54, 129.50, 130.30, 132.80, 132.92, 133.99, 136.02, 137.54, 139.54, 139.98, 141.85 (C_Ar_), 159.02, 177.59, 178.27 (C=O). LC-MS (70 eV): *m*/*z* = 467 [M + 1]^+^ (100%). Anal. Calcd. for C_21_H_9_BrClN_3_O_3_: C, 54.05; H, 1.94; N, 9.00. Found: C, 54.03; H, 1.97; N, 9.02.

*6-Bromo-3-(2-(trifluoromethyl)phenyl)anthra[1,2-d][1,2,3]triazine-4,7,12(3H)-trione* (**38**). Yield 75%; m.p.: 242 °C (decomposition); ^1^H-NMR (CF_3_COOD): *δ* = 7.66 (d, *J* = 7.8 Hz, 1H_Ar_), 7.72–7.78 (m, 1H_Ar_), 7.89 (s, 1H_Ar_), 8.04 (m, 3H_Ar_), 8.42 (d, *J* = 7.7 Hz, 2H_Ar_), 9.08 (s, 1H_Ar_). ^13^C-NMR (CF_3_COOD): *δ* = 119.52 (q, *J* = 32 Hz, C-CF_3_), 122.44, 123.81, 124.46, 125.32 (q, *J* = 273.05 Hz, CF_3_), 126.42, 126.91, 127.27, 127.98, 129.84 130.70, 131.48, 131.70, 132.38, 134.73, 135.26, 136.33, 139.54, 140.84 (C_Ar_); 154.87, 177.63, 178.24 (C=O). LC-MS (70 eV): *m*/*z* = 501 [M + 1]^+^ (100%). Anal. Calcd. for C_22_H_9_BrF_3_N_3_O_3_: C, 52.82; H, 1.81; N, 8.40. Found: C, 52.86; H, 1.83; N, 8.36.

### 3.3. Biology

#### 3.3.1. Bacterial Strains and Media

The antimicrobial potential of all synthesized compounds was evaluated against five reference strains of bacteria: *Staphylococcus aureus* ATCC 25923, *S. aureus* ATCC 29213, *S. epidermidis* ATCC 12228, *Pseudomonas aeruginosa* ATCC 27853, and *Escherichia coli* ATCC 25,922 and four reference strains of human pathogenic yeasts: *C. albicans* ATCC 10231, *C. albicans* SC5314, *C. krusei* DSM 6128 and *C. glabrata* DSM 11226. Because of the positive results of preliminary tests the anti-staphylococcal activity of selected agents was also investigated against eight MSSA and four MRSA clinical isolates derived from patients with different infections (Table 5). The antibiotics: ampicillin, oxacillin, gentamicin, fusidic acid, levofloxacin, linezolid, daptomycin were purchased from Argenda (Poznan, Poland). Bacteria were routinely grown on Luria-Bertani Agar (LA, Sigma Aldrich, Schnelldorf, Germany) and yeasts were cultivated on YPD Agar (A&A Biotechnology, Gdynia, Poland). In the case of bacteria, the Minimum Inhibitory Concentration (MIC) values were determined using Mueller-Hinton Broth 2 (MHB2, Sigma Aldrich) liquid medium—and determination of the same parameter for yeasts was performed using RPMI 1640 medium (Sigma Aldrich) supplemented with 2% glucose (Sigma Aldrich) and buffered to pH 7.0 with a MOPS buffer (3-*N*-morpholinopropanesulfonic acid) (Sigma Aldrich). For staphylococcal biofilm formation, TSB (Sigma Aldrich) liquid medium supplemented with 2.5% of glucose was used and Time-Kill assay was performed in Mueller-Hinton Broth 2.

#### 3.3.2. Investigation of Antimicrobial Potential of Synthesized Agents

The two-fold broth microdilution method, according to the CLSI standard methodology [42], was applied for the determination of minimum inhibitory concentrations (MICs) of synthesized agents and antibiotics against bacteria (both reference strains and clinical isolates). Bacterial strains were plated on Luria-Bertani Agar medium and incubated overnight at 37 °C. Two to three bacterial colonies were transferred from the agar medium into PBS buffer (pH = 7.4). The bacterial suspension was adjusted to the optical density of OD_600_ = 0.1 and diluted in MHB2 medium at a ratio of 1:100 *v*/*v* to the final cell concentration of approximately 0.5–1.0 × 10^6^ CFU/mL. The synthesized anthra[1,2-*d*][1,2,3]triazine-4,7,12(3*H*)-triones were dissolved in DMSO—final concentration of all substances tested in the stock solutions was 1280 µg/mL. The stock solutions with the same concentration (1280 µg/mL) of all antibiotics were used for investigation. Subsequently, a series of two-fold dilution of each agent in range 64.0–0.0625 µg/mL were prepared in the wells of columns 1–10 of 96-well plates using Mueller-Hinton Broth 2. An aliquot of 100 μL of inoculum was dispensed to the wells of columns 1–11. Column 11 contained 200 µL of inoculum without any agent, and column 12 contained 200 µL of the MHB2 broth only (served as a control of medium sterility). The plates were incubated 24 h under static conditions at 37 °C. The lowest concentration of agent with no visible bacterial growth was taken as a MIC value.

The determination of MIC values for yeast strains was also performed according to the appropriate NCCLS reference microdilution method [43]. The solutions of substances in DMSO were prepared in the same way as for the investigation of antibacterial activity (presented above). Serial two-fold dilutions of the tested substances were prepared in RPMI 1640 medium buffered to pH 7.0 with MOPS buffer (3-*N*-morpholinopropanesulfonic acid) in 96-well microtiter plates in a final volume of 100 µL. The final concentrations of the agents were in the range from 64 to 0.0625. Suspensions of the microorganisms were prepared by taking one loop of pure culture (cultivated 24 h at 37 °C on YPD agar) into sterile water and adjusting the optical density to 0.1 at 660 nm wavelength before further 50-fold dilution in an RPMI 1640 medium resulting in 2 × 10^4^ CFU/mL. One hundred microliters of such suspension were inoculated to each well of the microtiter plate, leaving a drug-free column (number 12) as sterility controls. Plates were incubated for 24 h at 37 °C. MIC values were read visually as the first concentration where no growth was observed. The MIC assay for each tested strain of bacteria/yeast and agent was performed in triplicate. In the case of both assays (investigation of antibacterial and antifungal activity) the final concentration of the solvent (DMSO) in the medium did not exceed 2.5% (*v*/*v*), and did not influence the growth of bacteria/yeast.

#### 3.3.3. Time-Kill Assay

Taking into account the results of previously performed analysis (high activity against staphylococci and lack of activity against Gram-negative bacteria and yeasts) the kinetic time-kill assay was performed for one reference strain of staphylococci (namely *S. aureus* ATCC 29213) and four, most promising, compounds (namely **32**, **33**, **34** and **36**). The suspension of approx. cell density 0.5–1.0 × 10^6^ CFU/mL of *S. aureus* ATCC 29,213 was prepared in MHB2 broth supplemented with a particular agent to the final concentrations equal to MIC, 2 × MIC or 4 × MIC and incubated at 37 °C with shaking (150 rpm). Bacterial suspension without agent addition was used as the untreated control. At predetermined time intervals (0, 1, 3, 6 and 24 h) samples were taken, serially diluted in PBS buffer (from 10^−1^ to 10^−7^) and spotted (10 µL) onto a Baird-Parker agar plate. After 24 h of incubation, the plates were enumerated. For each agent the assay was repeated in triplicate.

#### 3.3.4. Biofilm Formation and Determination of Antibiofilm Activity

The assay for biofilm cultivation was performed according to the procedure described previously [44] with slight modifications. The suspensions of approx. cell density 1–5 × 10^8^ CFU/mL of *S. aureus* ATCC 29,213 were diluted 1:100 (*v*/*v*) in TSB medium supplemented with 2.5% glucose. Cell suspensions (200 μL) were placed into the wells of columns 1–7 of vertically set plates. The wells of column 8 were fulfilled with 200 μL of sterile medium—these wells were used as a negative control. The plates were incubated for 24 h at 37 °C without shaking in order to allow bacteria to attach. In the next step, the formed biofilm was treated with four selected, most promising agents (**32**, **33**, **34** and **36**). The liquid medium was removed from the wells of the plates and the formed biofilm was gently washed with 200 μL of sterile PBS. Subsequently 200 μL of 2-fold serial dilutions of the agents in MHB2 medium, ranging from 64 to 2 µg/mL, were added to the wells of columns 1-6 (column 7—without agent but with formed biofilm served as positive control and wells of column 8—without biofilm, were used as negative control–control of sterility of the medium) and incubated for 24 h at 37 °C. The MTT assay was performed as described previously [44,45]. After 24-h treatment, the liquid content of the wells of the plates was removed and the treated biofilm was washed with 200 μL of sterile PBS buffer. Subsequently, 150 μL of PBS and 50 μL of MTT solution (0.3% in PBS) were added to the wells and mixed. Following 2 h incubation at 37 °C in the dark, the MTT solution was replaced with 200 μL of DMSO for dissolving the formed formazan crystals. The optical density of the obtained solutions was measured at 540 nm using a Victor^3^ microtiter reader (Perkin Elmer, Waltham, MA, USA). The Minimal Biofilm Eradiation Concentration (MBEC_50_) values were taken as the lowest concentration of the agent that caused eradication of at least 50% of living cells in comparison to the cells growing in the untreated control—measured as comparison of ability of living cells to the biotransformation of MTT (3-(4,5-dimethyl-2-thiazolyl)-2,5-diphenyl-2*H*-tetrazolium bromide) to insoluble in water violet formazan crystals [45].

#### 3.3.5. Influence of Investigated Agents on Enzymatic Activity of *S. aureus*

Fresh suspension (in PBS) of *S. aureus* ATCC 29,213 OD_600_ = 0.5 was exposed to the action of the selected tested agents (32, 33, 34 and 36) at concentration equal to ½ MIC for 1 h, 37 °C and then washed three times with PBS in order to avoid ‘carryover’ effect. Next, 65 microliters of the suspension were loaded to the cupules of the API ZYM strips. Suspension of untreated cells of *S. aureus* ATCC 29,213 served as controls. The strips API ZYM were incubated for 4 h at 37 °C and the results were read according to the manufacturer’s instructions. Enzymatic activity was determined in nanomoles of the hydrolyzed substrate according to the intensity of the color reaction on the scale 1–5, i.e., 1–5 nanomoles, 2–10, 3–20, 4–30, and 5–40 and more nanomoles. The intensity of the color was assessed visually.

## 4. Conclusions

A convenient method for the preparation of novel anthra[1,2-*d*][1,2,3]triazine-4,7,12(3*H*)-triones is proposed based on the diazotization reaction followed by cyclization of the corresponding 1-amino-9,10-dioxo-9,10-dihydroanthracene-2-carboxamides under acidic conditions at room temperature. The outcomes of our study revealed that some of the synthesized compounds (namely **32**–**34** and **36**) exhibit promising antistaphylococcal activity, which creates the prospect of their further in-depth studies as effective antibacterial agents.

## Figures and Tables

**Table 1:** Parameters of hydrogen bonds in crystal of compound 22.

D-H ···A	D-H(Å)	D···A(Å)	D-H-A(°)
N3-H3···O4	1.01(3)	2.732(3)	175 (3)
O4-H41··· O1_$1 *	0.92(5)	2.844(3)	166 (4)
O4-H42 ···O2_$2 *	0.91(5)	2.867(4)	142 (3)
O4-H42 ···N1_$2 *	0.91(5)	3.097(3)	143 (3)

* The atoms marked by symbol $1 and $2 are connected to base atoms by symmetry operations *1* − *x*, *3* − *y*, −*z* and *1 − x*, *y* + *0.5*, −*z* − *0.5*, respectively.

**Table 2:** Antimicrobial activity (MIC, μg/mL) of synthesized anthra[1,2-*d*][1,2,3]triazine-4,7,12(3*H*)-triones and randomly selected antibiotics.

Agent	Pathogen								
	*S. aureus* ATCC 25923	*S. aureus* ATCC 29213	*S. epidermidis* ATCC 12228	*E. coli* ATCC 25922	*P. aeruginosa* ATCC 27853	*C. albicans* ATCC 10231	*C. albicans* SC 5314	*C. glabrata* DSM11226	*C. krusei* DSM6128
22	4.0	4.0	8.0	>32.0	>32.0	>32.0	>32.0	>32.0	>32.0
23	2.0	2.0	1.0	>32.0	>32.0	>32.0	>32.0	>32.0	>32.0
24	0.5	0.5	0.5	>32.0	>32.0	>32.0	>32.0	>32.0	32.0
25	4.0	8.0	8.0	>32.0	>32.0	>32.0	>32.0	>32.0	>32.0
26	2.0	4.0	2.0	>32.0	>32.0	>32.0	>32.0	>32.0	>32.0
27	4.0	8.0	8.0	>32.0	>32.0	>32.0	>32.0	>32.0	>32.0
28	1.0	2.0	2.0	>32.0	>32.0	>32.0	>32.0	>32.0	>32.0
29	2.0	16.0	2.0	>32.0	>32.0	>32.0	>32.0	>32.0	>32.0
30	2.0	2.0	2.0	>32.0	>32.0	>32.0	>32.0	>32.0	>32.0
31	8.0	4.0	8.0	>32.0	>32.0	>32.0	>32.0	>32.0	>32.0
**32**	**0.25**	**0.25**	**0.125**	>32.0	>32.0	>32.0	>32.0	>32.0	>32.0
**33**	**0.25**	**0.25**	**0.25**	>32.0	>32.0	>32.0	>32.0	>32.0	>32.0
**34**	**0.25**	**0.5**	**0.25**	>32.0	>32.0	>32.0	>32.0	>32.0	>32.0
35	4.0	8.0	8.0	>32.0	>32.0	>32.0	>32.0	>32.0	>32.0
**36**	**0.25**	**0.5**	**0.125**	>32.0	>32.0	>32.0	>32.0	>32.0	>32.0
37	0.5	1.0	0.125	>32.0	>32.0	>32.0	>32.0	>32.0	>32.0
38	1.0	2.0	2.0	>32.0	>32.0	>32.0	>32.0	>32.0	32.0
Ampicillin	0.25	8.0	16	>32.0	>32.0	ND	ND	ND	ND
Gentamicin	0.125	0.5	0.5	2.0	2.0	ND	ND	ND	ND
Fusidic acid	0.5	0.25	0.125	>32.0	>32.0	ND	ND	ND	ND
Linezolid	2.0	1.0	1.0	>32.0	>32.0	ND	ND	ND	ND
Daptomycin	1.0	1.0	2.0	>32.0	>32.0	ND	ND	ND	ND
Oxacillin	0.25	0.125	0.125	>32.0	>32.0	ND	ND	ND	ND
Levofloxacin	0.125	0.25	0.125	>32.0	>32.0	ND	ND	ND	ND

Eight substances selected for investigation of their activity against clinical isolates are marked with a gray background and four compounds with the highest activity that were selected for further study (kill-time assay, antibiofilm activity, and influence on enzyme activity) are underlined and bolded. ND—not determined.

**Table 3:** Antimicrobial activity (MIC, μg/mL) of eight selected compounds against clinical isolates of *S. aureus* (MSSA and MRSA).

Agent	Pathogens—Clinical Isolates of *S. aureus*											
	1	2	3	4	5	6	7	8	MRSA1	MRSA2	MRSA3	MRSA4
23	4.0	8.0	4.0	8.0	8.0	8.0	8.0	8.0	8.0	2.0	8.0	8.0
24	1.0	1.0	0.5	0.25	0.25	1.0	0.5	0.5	1.0	1.0	0.5	0.5
26	2.0	2.0	1.0	1.0	1.0	4.0	1.0	2.0	4.0	2.0	1.0	2.0
32	0.25	0.25	0.25	0.25	0.25	0.5	0.25	0.125	0.25	0.25	0.25	0.25
33	0.5	0.5	0.5	0.5	0.5	0.5	0.5	0.5	0.5	0.5	0.25	0.5
34	0.25	0.5	0.25	0.25	0.5	0.25	0.25	0.25	0.5	0.125	0.25	0.25
36	0.25	0.5	0.25	0.5	1.0	0.5	0.25	0.5	0.5	1.0	0.25	0.5
37	0.5	0.5	0.5	1.0	1.0	0.5	1.0	0.5	1.0	1.0	1.0	2.0

**Table 4:** Influence of selected compounds on enzymatic activity of *S. aureus* ATCC 29,213.

Compound	Enzyme				
	Alkaline Phosphatase	Esterase (C4)	Esterase Lipase (C8)	Acid Phosphatase	Naphthol-AS-BI-Phosphohydrolase
Control	5	5	5	5	5
**32**	4	5	4	5	5
**33**	3	4	4	5	5
**34**	3	3	4	5	5
**36**	4	2	4	5	5

**Table 5:** MSSA and MRSA strains used for investigation of antistaphylococcal potential of synthesized agents.

No.	Code of the Strain	Material	Ward of Hospital	Antibiogram—Susceptibility Profile ^1^
1	4471313	Nasal swab	Intensive care	Pen.—R, Met.—S, Clin.—S, Ery.—S
2	4475564	Nasal swab	Internal	Pen.—R, Met.—S, Clin.—R, Ery.—R
3	4476206	Sputum	Internal	Pen.—R, Met.—S, Clin.—R, Ery.—R
4	4475131	Pus	Internal	Pen.—R, Met.—S, Clin.– R, Ery.—R
5	4466686	Sputum	Surgical	Pen.—R, Met.—S, Clin.—R, Ery.—R
6	4466380	Wound	Surgical	Pen.—R, Met.—S, Clin.—S, Ery.—S
7	4466896	Nasal swab	Internal	Pen.—R, Met.—S, Clin.—S, Ery.—S
8	4468792	Pharyngeal swab	Pediatrics	Pen.—R, Met.—S, Clin.—S, Ery.—S
9-MRSA	9572250	Wound	Internal	Pen.—R, Met.—R, Clin.—R, Ery.—R
10-MRSA	8007171	Wound	Laryngology	Pen.—R, Met.—R, Clin.—R, Ery.—R
11-MRSA	45300223	Blood	Pediatrics	Pen.—R, Met—R, Clin.—R, Ery.—R
12-MRSA	9935169	Wound	Dispensary	Pen.—R, Met.—R, Clin.—R, Ery.—R

^1^ Identification of bacterial isolates and antibiograms were performed by Laboratory of Clinical Microbiology, University Centre for Laboratory Diagnostics, Medical University of Gdańsk Clinical Centre with Vitek2 Biomerieux system; Pen—Penicillin, Met—Methicillin, Clin—Clindamycin, Ery—Erythromycin, R—resistant, S—sensitive.
